# REFINING AN ELDER MISTREATMENT SCREENING AND RESPONSE TOOL TO IMPROVE ACCEPTABILITY IN AN EMERGENCY DEPARTMENT

**DOI:** 10.1093/geroni/igz038.290

**Published:** 2019-11-08

**Authors:** Brian Tanksley, Theresa Sivers-Teixeira, Laura Mosqueda, Bonnie Olsen, Tim Platts-Mills, Kristin Lees-Haggerty

**Affiliations:** 1 USC Keck School of Medicine, Los Angeles, California, United States; 2 Keck School of Medicine USC, Alhambra, California, United States; 3 Keck School of Medicine USC, Los Angeles, California, United States; 4 University of North Carolina at Chapel Hill, Chapel Hill, North Carolina, United States; 5 Education Development Center, Waltham, Massachusetts, United States

## Abstract

Elder mistreatment (EM) is a public health problem that is rarely recognized or addressed in emergency departments (ED) where a lack of evidence-based protocols leave clinicians to rely on intuition and inconsistent action plans. In this presentation we will share findings from focus groups and online surveys with ED clinicians and administrators to evaluate the perceived value and likelihood of adopting the National Collaboratory’s third core element: the EM Screening and Response Protocol (EM-SAR). Results indicated a strong support for the EM-SAR tool in general and highlighted specific considerations for refining the tool. Considerations include resistance to adding to the ED workload, need to clarify roles and responsibilities for administering the tool, hesitancy to rely on clinical judgement to assess EM, concerns over Adult Protective Services’ ability to respond to increased reports, and a desire for cross-training and cooperation. These findings and implications for ongoing feasibility testing will be discussed.

